# Longitudinal relationship between depression and antisocial behaviors in Korean adolescents

**DOI:** 10.3389/fpsyt.2023.1053759

**Published:** 2023-06-02

**Authors:** Na Ri Kim, Yeong Seon Jo, Young Il Cho, Younyoung Choi, Sang Jin Park

**Affiliations:** ^1^Graduate School of Education, Ajou University, Suwon, Republic of Korea; ^2^Department of Psychiatry, Seoul St. Mary's Hospital, College of Medicine, The Catholic University of Korea, Seoul, Republic of Korea; ^3^Division of Police Administration, Dongguk University, Seoul, Republic of Korea; ^4^Department of Psychology, Ajou University, Suwon, Republic of Korea; ^5^Department of Transdisciplinary Security, Dongguk University, Seoul, Republic of Korea

**Keywords:** Korean adolescents, delinquency, depression, sex difference, autoregressive cross-lagged model, multi-group analysis, failure model, acting-out model

## Abstract

**Background:**

It is well known that depression and delinquency in adolescents are highly correlated, but longitudinal studies on the causal relationship between them are not active in East Asia compared to in Western culture. In addition, even the results of research on causal models and sex differences are inconsistent.

**Objectives:**

This study examines the longitudinal reciprocal effects between depression and delinquent behavior in Korean adolescents based on sex differences.

**Methods:**

We conducted multiple-group analysis by using an autoregressive cross-lagged model (ACLM). Longitudinal data from 2,075 individuals (2011–2013) were used for analysis. The longitudinal data are from the Korean Children and Youth Panel Survey (KCYPS), and data were used beginning with students at 14 years old (in the second grade of middle school) and tracked them until they were 16 (in the first grade of high school).

**Results:**

Boys’ delinquent behaviors at 15 years (the third grade of middle school) affected their depression at 16 years (the first grade of high school). In contrast, girls’ depression at 15 years (the third grade of middle school) influenced their delinquent behaviors at 16 years (the first grade of high school).

**Discussion:**

The findings support the failure model (FM) among adolescent boys and the acting-out model (ACM) among girls. The results imply that strategies to effectively prevent and treat delinquency and depression in adolescents must consider sex effects.

## 1. Introduction

Adolescence is a phase of developmental trajectories characterized by confusion and conflict resulting from multifaceted transitions, including physical, emotional, and behavioral changes. Many adjustment-related challenges during this period fall into two categories: (a) internalizing disorders comprising emotional problems such as depression and anxiety; and (b) externalizing disorders, where inner conflicts are acted out through aggressive behaviors and delinquencies (1). Depression, the representative internalizing disorder in adolescence, is due to factors such as brain maturity, academic stress, interpersonal sensitivity, and emotional instability that increase during adolescence (2–4). Depressive disorders are the most prevalent of all mental disorders and are found in 5–12% of people worldwide depending on race, country, and evaluation methods (5). A recent study of adolescents showed that the prevalence of major depression disorder (MDD) and dysthymia was 8 and 19%, respectively, and depression symptoms increased by 24% between 2001 and 2010 and by 37% between 2011 and 2020 (6). Depression in this period is the prime factor for causing self-harm and suicide (7, 8), academic difficulties (9), and is also highly associated with other mental disorders such as anxiety, drug abuse, and behavioral disorders (10). Delinquency, a representative phenomenon of adolescents’ externalizing disorders, is medically diagnosed as conduct disorder, found in 6–18% of men and 2–9% of women under the age of 18, and its prevalence has risen in recent years (11). Parental coerciveness, inconsistent discipline, low socioeconomic status, and community violence are strong risk factors for behavioral disorders (12), and adolescents with behavioral disorders face serious problems such as low academic achievement, family dysfunction, drug misuse, emotional distress, suicide, and criminal involvement (13, 14).

Internalizing and externalizing disorders manifest in remarkably dissimilar ways, but they often coexist (15, 16). Depression and delinquency, the representative psychological disorders in each category, are the most frequently detected problems in adolescence, and there are numerous investigations on their comorbidity (17–20). According to Greene et al. (21), more than 30% of adolescents with depression disorders also have behavioral disorders, and more than 50% of adolescents who meet the criteria for behavioral disorders also match the criteria for depression. In addition, the rate of comorbidity between depression and aggression in children and adolescents varies from 8 to 11% in the general population (22, 23) to 24% in the clinical population (24). These studies found higher maladjustment, including lower academic performance, interpersonal conflicts, lower self-efficacy, substance abuse, and an unfavorable prognosis in comorbidity than in single-morbidity cases (25–27).

The potential severity of outcomes associated with depression-delinquency comorbidity has prompted significant efforts to develop prevention and treatment strategies based on its causes and patterns. In earlier phases of this research, comorbidity data were analyzed using cross-sectional approaches (28, 29). However, these approaches cannot explain the co-occurrence of depression and delinquency manifesting over time. Additionally, cross-sectional techniques are limited as they cannot identify the temporal precedence of the two disorders occurring at different time points (19). More recently, numerous longitudinal studies of depression-delinquency comorbidity have led to theoretical models addressing its developmental mechanisms, including failure models (FM), acting-out models (AOM), and reciprocally related cyclical patterns (RRCP) (30).

The FM explains various failures associated with delinquent behaviors, including lower self-efficacy, conflicts with parents, rejection of and by significant role models and peers, and poor academic achievement, which ultimately cause depression (31–33). In the U.S., Capaldi (31) investigated the stabilization of depression and conduct disorders (CD) over the course of 2 years among sixth-grade boys, finding that their delinquencies predicted higher depression in the eighth grade, while their depression failed to predict increased delinquencies. Capaldi’s findings were supported by many studies. For example, a comorbidity study of American adolescents aged 11 to 14 revealed that the diagnosis of disruptive behavior disorder preceded that of depression (34). Another cohort study of American children aged 7 to 12 tracked the subjects until they reached the age of 18, revealing that their CD symptoms predicted their depression in later years, whereas their depression failed to predict their CD (35). Similarly, a study of Korean adolescents (first grade in middle school) analyzed the longitudinal data over a period of 3 years and found that delinquencies significantly predicted the changes in the rates of depression both in boys and girls (36). A study by Lee and Oh (19) tracked 13–14-year-old boys (from the first to second grade of middle school) for a year and a half and confirmed the same findings, thus supporting the FM.

The AOM, which theorizes that depression precedes delinquency, also has some empirical support. Wiesner (37) conceptualized masked depression to explain behavioral problems, including delinquencies, where they are believed to mask underlying depression. Additionally, Puig-Antich (29) reported that 87% of American children with dual depression and CD diagnoses developed depression first and that depression treatment decreased their behavioral problems. Other studies of American adolescents have also supported the AOM. Beyers and Loeber (38) used a sample of adolescents aged 13 to 17 and reported that depression in this period significantly predicted antisocial behaviors in later years. Childhood major depressive disorder (MDD) or bipolar disorder was found to predict delinquent behaviors in adolescence (39). In another study, symptoms of depression were found to predict antisocial behaviors in later years, whereas the findings did not validate the opposite conclusion (40). Additionally, a longitudinal study of Korean middle school students in Grade 2 analyzed the data accumulated over a period of 5 years and discovered that depression in the earlier years of the research period influenced the changes in delinquencies, but delinquencies failed to influence the occurrences of depression (41).

The RRCP was proposed to reconcile the conflicting FM and AOM concepts (37). Unlike the FM and AOM, which assume a unidirectional relationship between depression and delinquency, RRCP assumes that the two morbidities are simultaneously risk factors and outcomes, supporting a bidirectional relationship. However, previous studies found that the extents to which depression and problem behaviors influence each other were asymmetrical and that the rate at which delinquency increased depression far outpaced that at which depression increased delinquency (42, 43). Measelle et al. (44) analyzed data from 493 American preadolescent girls and reported that an increase in depression or delinquency predicted an increase in the respective comorbidities. Other studies have also supported a bidirectional relation between delinquency and depression by investigating the morbidities in 15–16-year-old American female students (37, 45). A study of Korean preadolescent students (ages 10 to 13) revealed that depression in earlier years affected depression and delinquency in later years, and delinquency in earlier years affected delinquency and depression in later years, respectively (46). Furthermore, a bidirectional relationship was found to exist between depression and delinquency in middle school girls (43). Numerous subsequent studies have supported a bidirectional relationship between delinquency and depression (45, 47), validating the RRCP.

The studies that have been reviewed so far regarding the association between depression and delinquency have mostly been conducted in Western cultures. Therefore, it would be possible to compare the differences between the two cultures by conducting the same study on Eastern culture. As a result of a comparative study of educational backgrounds among more than 40 countries, Korea showed relatively high achievement among them in terms of the proportion of top-ranked students as well as the average achievement level (48). In the background of such high achievement, there is an excessively competitive learning atmosphere, and due to this, Korean adolescents have low academic interest or autonomy, while academic stress and test anxiety are high (49). This phenomenon is common in Eastern cultures, such as Taiwan and Japan (49). These academic-related variables are highly related to depression or delinquency in Eastern cultures (50, 51). Additionally, since academic stress is known to bring emotional and behavioral problems in Western cultures (52, 53), the results of this study provide the opportunity to compare empirically the associations among the variables between the two cultures.

Specific relationships between the two morbidities vary, resulting in inconsistent results across studies. This suggests a potential difference in the relationship between depression and delinquency concerning one’s sex. Since there is a sex difference in each of the two variables, it may affect the relationship between them. Notably, studies show that adolescent boys reflect the FM, whereas adolescent girls reflect the RRCP (43). Additionally, it is noteworthy that Western countries have actively conducted longitudinal data-based research, whereas East Asian countries have not. Furthermore, even a small number of East Asian studies have delivered conflicting results supporting different models. Therefore, it is necessary to continue to research Eastern culture; by doing so, it will be revealed whether reciprocal relationships between depression and delinquency are similar or different depending on the culture. Previous studies have demonstrated inconsistent results for the presence or absence of sex differences. This study examines the order in which depression and delinquency develop in Korean adolescent boys and girls, focusing on changes during a 3-year period beginning at 14 years old (Y2: second grade of middle school) and ending at 16 years old (Y4: first grade of high school). In the Republic of Korea, as tests and evaluations begin at 14 years old (Y2: second grade of middle school), emotional difficulties and identity confusion are experienced due to the pressure of academic achievement and rapidly increasing private education (54). These 3 years also coincide with dramatic increases in stress concerning acceptance into high school (55), which leads to significant changes in Korean adolescents and potentially underlies various adolescent adjustment problems.

Therefore, we aim to verify the causal direction between depression and delinquency in Korean adolescents at three points (Y2–Y4: 14–16 years) by applying the autoregressive cross-lagged model (ACLM). In addition, we examine the difference between boy and girl students by comparing the causal direction according to sex. In summary, this study conducted a multi-group analysis by applying ACLM to longitudinal relationships between depression and delinquency according to sex to investigate their developmental causes and identify relationships between the two morbidities. The research questions of this study are as follows. First, what is the relationship between depression and delinquency among adolescents? Second, what is the change in depression and delinquency among adolescents according to the passage of time? Third, is there any difference in the causal direction of depression and delinquency according to the passage of time between male and female students?

## 2. Methods

### 2.1. Participants and procedure

**Figure 1:**
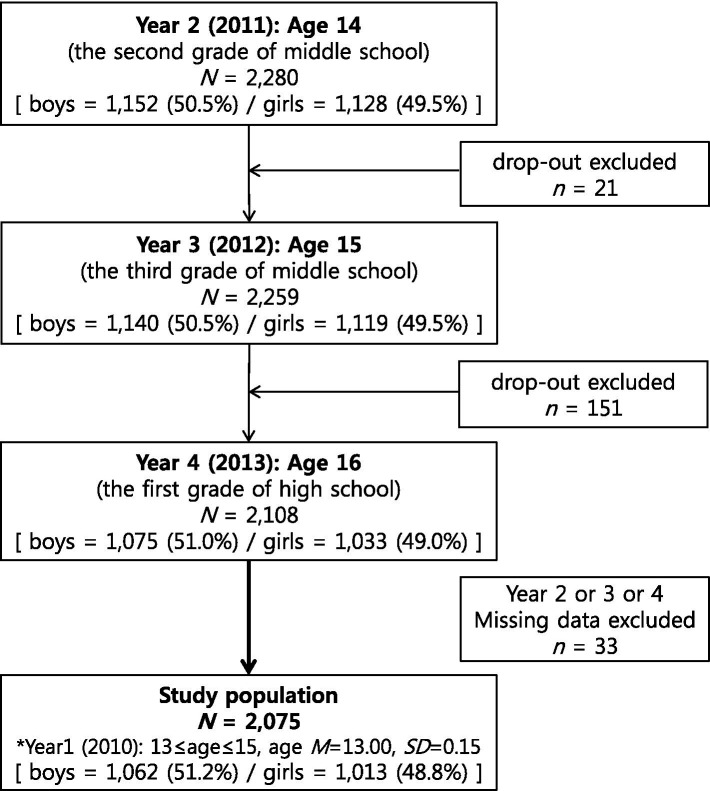
Participants flow chart.

The Korea Children and Youth Panel Survey (KCYPS) is an open data collection provided by the National Youth Policy Institute (NYPI) for children and adolescents in Korea (56). We used KCYPS longitudinal data (2010–2016) from 13 to 15-year-old students who were in their first grade of middle school nationwide as of 2010 (Y1), with permission and approval from NYPI. Participants were then sampled using stratified multistage cluster sampling. Among the longitudinal data, we used three-year data from 2011 to 2013, the period when depression and delinquency were measured in this study. Specifically, the Y2 sample (2011: second grade of middle school) contained 2,280 students (1,152 boys, 50.5%; and 1,128 girls, 49.5%), which decreased to 2,259 (1,140 boys, 50.5%; and 1,119 girls, 49.5%) in the Y3 sample (2012: third grade of middle school). In Y4 (2013: first grade of high school), 151 students were lost, which left 2,108 participants (1,075 boys, 51.0%; and 1,033 girls, 49.0%). After excluding questionnaires with missing values, a total of 2,075 samples containing all data from 2011 to 2013 were used for analysis. At the beginning of the survey (Y1, 2010), the average age of the 2,075 students was 13.00 (*SD* = 0.15), and 1,062 boys (51.2%) and 1,013 girls (48.8%) were included in the analyses (see Figure 1). Therefore, Y2 (2011) was 14 years old (second grade of middle school), Y3 (2012) was 15 years old (third grade of middle school), and Y3 (2013) was 16 years old (first grade of high school).

### 2.2. Measures

Panel data collection was conducted through individual face-to-face surveys by the NYPI. Data were collected by the adolescents responding to a self-report form after a surveyor visited and briefly explained the writing method.

#### 2.2.1. Depressive mood

The Symptom Checklist-90-Revised (SCL-90-R) developed by Derogatis (1977) was validated to fit the situation in Korea (57). Among the nine symptom scales, the depression scale was used, and a total of 10 items were investigated, excluding 3 items from the 13 items. The following ten items measured depression: (1) I do not have much energy; (2) I feel unhappy, sad, or depressed; (3) I have a lot of worries; (4) I feel like dying; (5) I often cry; (6) When things go wrong, I often think it is because of me; (7) I am lonely; (8) I do not have interest in or enthusiasm for anything; (9) I do not see much of a future for me; and (10) Everything is hard. Each item used a 4-point Likert scale (1 = strongly disagree, 4 = strongly agree), where higher total scores indicated higher depression levels. In our sample, internal consistency reliability was 0.901, 0.906, and 0.889 at 14 years old (Y2: second grade of middle school), 15 years old (Y3: third grade of middle school), and 16 years old (Y4: first grade of high school), respectively.

Exploratory factor analysis (EFA) was conducted to identify depression characteristics and construct item parcels, with results indicating a one-factor model. Accordingly, items were allocated to achieve similar means for the factor loading of latent variables, and three parcels were constructed (58). Of the 10 depression items, “Depression 1” was allocated three items (nos. 10, 6, and 4), “Depression 2” three items (nos. 2, 9, and 3), and “Depression 3” four items (nos. 7, 8, 1, and 5).

#### 2.2.2. Delinquent

The questions of the Korean juvenile delinquency scale developed by Kim and Lee (59) were used. Thirteen yes-or-no items were used to investigate participants’ delinquent behavior experiences, as follows: (1) smoking; (2) drinking; (3) truancy; (4) running away from home; (5) excessive teasing or mocking others; (6) ostracizing others; (7) participating in group fights; (8) beating others; (9) threatening others; (10) extorting (money or things); (11) stealing (money or things); (12) engaging in sexual relationships; and (13) taking part in sexual assault or harassment.

The delinquency characteristics were identified using EFA, and item parcels were then constructed. Prior to the factor analysis, the sampling suitability of the delinquency scales was examined with the Kaiser–Meyer–Olkin (KMO) test and Bartlett’s sphericity test. The results showed that the KMO value (where a value closer to 1 indicates an adequate sample for factor analysis) was 0.743 (60, 61). The results of Bartlett’s test revealed *χ*^2^ = 3393.314, *df* = 78, and *p* < 0.001, rejecting the null hypothesis that the data do not include the factor structures.

Next, eigenvalues in the scree plot were confirmed (60, 62). A four-factor model was suggested by the results, and the four factors were named through a content-based approach (63, 64). The 13 delinquency items were divided into the following: 4 items for status delinquency (nos. 1, 2, 3, and 4); 5 items for violence delinquency (nos. 5, 6, 7, 8, and 9); 2 items for property delinquency (nos. 10 and 11); and 2 items for sex delinquency (nos. 12 and 13). As these are categorical judgment items, the delinquency factor value was the sum of the means for the four factors, where higher scores indicated higher delinquency. In our sample, internal consistency reliability values were low at 0.684, 0.652, and 0.569 at 14 years old (Y2: second grade of middle school), 15 years old (Y3: third grade of middle school), and 16 years old (Y4: first grade of high school), respectively. However, notably, the delinquency variable comprises formative indicators (status, violence, property, and sex), where higher levels of internal consistency and reliability can be problematic for collinearity. Therefore, the correlation may be low (65).

### 2.3. Statistical analysis

We used ACLM to test the hypotheses. Unlike cross-sectional models that cannot identify temporal precedence between variables, ACLM verifies bidirectional relationships in longitudinal data, thereby identifying causal directions (66). ACLM expands the autoregressive model, which explains the value for a particular time point (*t*) from the value of a previous time point (*t*-1) (67), allowing for the prediction of auto-regression effects and cross-lagged effects that demonstrate the reciprocal relationships between variables (66). The model allows for the demonstration of changes that occur in the influence of one variable on others over time. Specifically, researchers control the effects of delinquent behavior at a previous time point and then examine relations, if any, between depression at a previous time point and delinquent behavior at a later time point. Reversely, the effects of depression at an earlier time point are controlled, and then any relations between delinquency at an earlier time point and depression at a later time point are investigated longitudinally. Such examination allows for the identification of temporal precedence.

Multi-group analysis was performed using Mplus version 8.0 for the ACLM analysis. IBM SPSS Statistics version 23.0 was used for descriptive statistics and correlation analysis. First, the measurement model was verified by parceling items based on subscales or EFA results and the construct of three or four measurement variables for each latent variable. Next, metric measurement invariance was verified. Therefore, it was confirmed that the same measurement model for latent variables can estimate differences between groups at the latent variable level based on the intra-group differences at the measurement variable level. Specifically, this study aimed to compare the regression coefficients between depression and delinquency. Thus, the configural and metric invariances for the depression measurement model were examined sequentially. Configural invariance tests whether the construct structures are the same between time points and between sex groups. Metric invariance verifies whether the same units are applied to the measurement at each time point and across sex groups. After establishing the configural and metric invariances, congruence coefficients for the auto-regression model (i.e., the structural model for depression and delinquency) were tested. In doing so, ACLM was examined for sex differences. To handle the non-normality of delinquency measures, the alternative of the maximum likelihood (ML) estimation method was employed (68–71). Specifically, maximum likelihood with robust standard errors (MLR) is a commonly used robust estimator of estimates and their standard errors, improving accuracy and handling of the non-normality of the measures.

The chi-squared test (*χ*^2^) was used to test the congruence of the model, and the Tucker–Lewis index (TLI), comparative fit index (CFI), and root mean square error of approximation (RMSEA) were applied to evaluate the model fit. Notably, the models adopted for this study are nested ones. Thus, intra-model comparisons used *χ*^2^ tests. Due to its sensitivity to sample sizes, TLI, CFI, and RMSEA differences were examined as well. There is a significant difference between the two models when the value of *p*s for their ∆*χ*^2^ are significant. What this means is that the more complicated of the two models can better account for the data than the less complicated one (72). On the contrary, a *value of p* that is not significant for ∆*χ*^2^ would mean that there are no significant differences between the two models. In that case, the simpler of the two models is considered to better account for the data. Furthermore, model congruence is interpreted such that it is significantly worse when TLI decreases to or below 0.02 (73), or when CFI decreases to or below 0.01 (74). RMSEA, on the other hand, is considered an indication of significantly worse model congruence when the value increases to or beyond 0.015 (75). In other words, the more complicated model of the two better accounts for the data than the less complicated one. In the other case, the model congruence was not significantly worse, and the more parsimonious model is understood to better account for the data.

## 3. Results

### 3.1. Depression and delinquency descriptive statistics and correlations

Table 1 presents the adolescence depression and delinquency descriptive statistics and correlation analysis for each time point. Depression at 15 years old (Y3: third grade of middle school) increased slightly from 14 years old (Y2: second grade of middle school) but decreased at 16 years old (Y4: first grade of high school) to a level below when they were 14 (Y2: second grade of middle school). Sex delinquency was consistently low. Status delinquency increased annually, whereas violence and property delinquency decreased over time. Over the three-year period, overall significant correlations were observed between depression and delinquency.

**Table 1:** Correlation of variables among middle school 1 panel (*N* = 2,075).

		Age 14 (2011: Middle school 2)							Age 15 (2012: Middle school 3)							Age 16 (2013: High school 1)						
		1	2	3	4	5	6	7	1	2	3	4	5	6	7	1	2	3	4	5	6	7
Age 14 (2011: Middle school 2)	1	1																				
	2	0.777^**^	1																			
	3	0.786^***^	0.766^***^	1																		
	4	0.111^***^	0.076^**^	0.059^**^	1																	
	5	0.059^**^	0.052^*^	0.039	0.286^***^	1																
	6	0.018	0.018	0.016	0.356^***^	0.379^***^	1															
	7	0.026	0.054^*^	0.045^*^	0.117^***^	0.078^***^	0.184^***^	1														
Age 15 (2012: Middle school 3)	1	0.455^***^	0.417^***^	0.036	0.063^**^	0.009	0.015	−0.014	1													
	2	0.412^***^	0.448^***^	0.412^***^	0.060^**^	0.033	0.034	0.026	0.776^***^	1												
	3	0.409^***^	0.402^***^	0.418^***^	0.037	0.003	0.005	0.011	0.794^***^	0.768^***^	1											
	4	0.060^**^	0.026	0.449^***^	0.507^***^	0.177^***^	0.263^***^	0.052^*^	0.122^***^	0.082^***^	0.072^***^	1										
	5	0.041	0.045^*^	0.025	0.136^***^	0.239^***^	0.160^***^	0.016	0.025	0.033	0.008	0.221^***^	1									
	6	0.065^**^	0.032	0.017	0.209^***^	0.134^***^	0.188^***^	−0.004	0.059^**^	0.043	0.002	0.282^***^	0.408^***^	1								
	7	−0.004	0.012	0.030	0.035	0.039	0.036	0.233^***^	0.047^*^	0.061^**^	0.045^*^	0.106^***^	0.034	0.118^***^	1							
Age 16 (2013: High school 1)	1	0.343^***^	0.318^***^	0.011	0.077^***^	0.024	−0.017	0.009	0.434^***^	0.360^***^	0.390^***^	0.089^***^	0.049^*^	0.035	0.004	1						
	2	0.337^***^	0.353^***^	0.313^***^	0.064^**^	0.056^*^	0.001	0.015	0.400^***^	0.430^***^	0.400^***^	0.062^**^	0.048^*^	0.025	0.021	0.749^***^	1					
	3	0.346^***^	0.352^***^	0.323^***^	0.072^**^	0.062^**^	0.002	0.021	0.419^***^	0.419^***^	0.466^***^	0.080^**^	0.014	0.029	0.016	0.767^***^	0.746^***^	1				
	4	0.031	0.004	−0.011	0.349^***^	0.177^***^	0.179^***^	0.053^*^	0.086^***^	0.042	0.022	0.465^***^	0.066^**^	0.131^***^	0.041	0.103^***^	0.062^**^	0.057^**^	1			
	5	0.037	0.028	0.010	0.113^***^	0.177^***^	0.111^***^	−0.009	0.021	0.005	−0.020	0.079^***^	0.111^***^	0.144^***^	−0.013	0.014	0.005	−0.007	0.214^***^	1		
	6	0.037	0.007	0.025	0.076^***^	0.043^*^	0.069^**^	−0.003	0.063^**^	0.061^**^	0.029	0.057^**^	0.008	0.156^***^	−0.004	0.027	0.015	0.018	0.091^***^	0.285^***^	1	
	7	0.033	0.021	0.011	0.018	0.021	0.033	−0.003	0.028	0.014	0.002	0.074^**^	−0.013	0.053^*^	0.101^***^	0.061^**^	0.041	0.052^*^	0.121^***^	0.037	−0.004	1
*M*		1.85	2.02	1.90	0.04	0.03	0.01	0.00	1.92	2.06	1.98	0.05	0.01	0.01	0.00	1.78	2.01	1.86	0.07	0.01	0.00	0.00
*SD*		0.658	0.707	0.625	0.134	0.102	0.081	0.031	0.682	0.704	0.649	0.144	0.063	0.059	0.033	0.591	0.664	0.586	0.163	0.056	0.038	0.035

*p < 0.05, **p < 0.01, ***p < 0.001. 1, depression1; 2, depression2; 3, depression3; 4, status delinquency; 5, violent delinquency; 6, property delinquency; 7, sex delinquency.

### 3.2. ACLM-applied multi-group analysis of relationships between depression and delinquency based on sex

#### 3.2.1. Testing the depression measurement model for students aged 14–16 (Y2–Y4) based on sex

**Figure 2:**
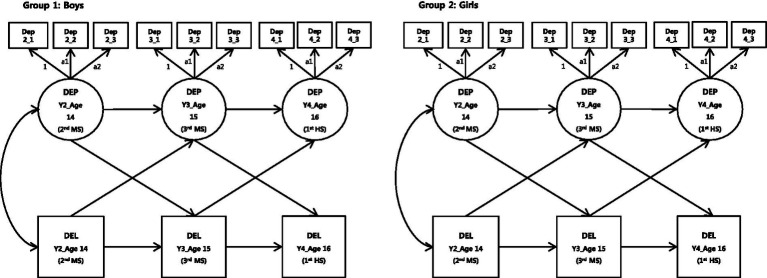
ACLM for depression and delinquency in Y1 boys and girls.

Figure 2 shows an ACLM model of depression and delinquency in male and female students. First, models were defined sequentially to verify measurement invariance for depression. Model 1 was a baseline model subject to configural invariance across sex groups to test the congruence of constructs and structures between males and females. Model 2 tested whether males and females responded similarly to items at each time point. Hence, this model added metric invariance to Model 1 for each sex across time points. Figure 2 lists the specific information about Model 2, in which factor loading was allocated equally to three time points for sex-specific depression measurements. Males were assigned to 1, a1, and a2 for the three time points, and females were assigned to 1, b1, and b2. Model 3 added metric covariance to Model 2 across sex groups and tested whether the same measurement units were applied to both males and females. The model allocated the same factor to both sexes, loading 1, a1, and a2 at three time points for depression measurements.

A comparison of the congruence coefficient across the three models showed that the hypotheses for both the configural and metric covariances were confirmed (see the model fit indices in Table 2). Models 1 and 2 had statistically significant *p*-values for ∆*χ*^2^ (∆*χ*^2^ = 17.746, ∆*df* = 8, *p* = 0.02), demonstrating significant differences between the two models (72). However, the possibility of sample size effects cannot be ruled out, considering the number of cases included in the study (*N* = 2,075). Other congruence coefficients were also checked. The TLI values between the models exceeded 0.02 (73), and the CFI values exceeded 0.01. Therefore, the congruence across the models was suitably maintained (74). Moreover, the RMSEA values between the model pairs exceeded 0.015, showing adequate congruence across the models (75). Based on the results, the metric covariance at each time point and between sex groups for the depression measurement models was established. Model 3, the most parsimonious of the three, was selected as the final model. It verified the hypotheses that the construct structures at each time point and between sex groups are congruent, and the participants responded to the survey items in a similar fashion. This allowed for the next step, where the ACLM for depression and delinquency was compared across sex groups.

**Table 2:** Testing models’ measurement invariance for depression (model fit).

Model	Study population (*N* = 2,075)								
	*χ*^2^	*df*	TLI	CFI	RMSEA [90%CI]	∆*χ*^2^ (∆*df*)	∆TLI	∆CFI	∆RMSEA
1	391.240***	48	0.963	0.975	0.083 [0.076, 0.091]				
2	408.986***	56	0.967	0.975	0.078 [0.071, 0.085]	17.746 (8)	0.003	0	−0.005
3	412.214***	58	0.968	0.974	0.077 [0.070, 0.084]	3.228 (2)	0.001	−0.001	−0.001

*p < 0.05, **p < 0.01, ***p < 0.001.

#### 3.2.2. ACLM for depression and delinquency in boys and girls aged 14–16 (Y2–Y4)

The congruence coefficients for the final model (Model 3) were tested (ACLM for sex-specific depression and delinquency), yielding significant results (*χ*^2^ = 539.181, *df* = 102, *p* < 0.001). However, *χ*^2^ is sensitive to sample sizes. Accordingly, the TLI, CFI, and RMSEA results were examined as well: TLI = 0.962, CFI = 0.971, RMSEA = 0.064 [0.059–0.070]. The TLI and CFI values were more than 0.95 (76), and the RMSEA value was less than 0.08. Therefore, Model 3 was confirmed as a good fit for the data (77, 78).

**Figure 3:**
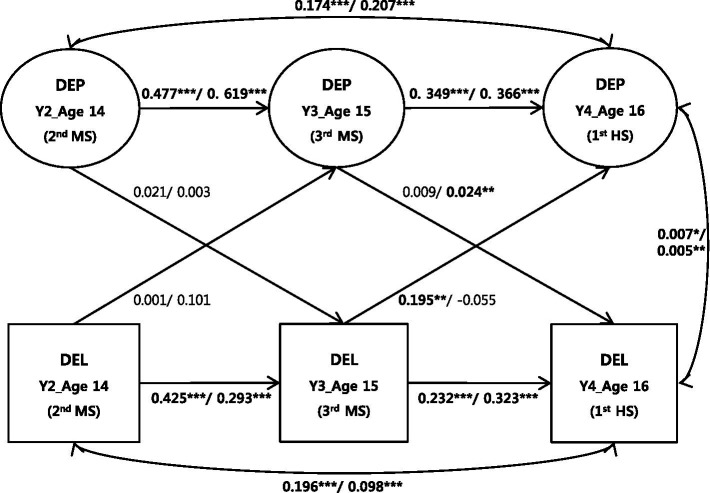
Path coefficient estimates for ACLM for depression and delinquency in boys and girls (Y1). Values indicate unstandardized coefficients for boys (left of/) and girls (right of/). DEP, depression; DEL, delinquency; G, grade; MS, middle school; HS, high school.

Table 3 and Figure 3 present the results of testing ACLM for depression and delinquency in males and females. First, the autoregressive coefficients were statistically significant for depression and delinquency in both sexes, indicating that adolescent depression and delinquency continue to influence the two morbidities in later years, regardless of sex. In other words, both sexes experience stable maintenance of the two morbidities over time. Next, time-based reciprocal relationships between the two morbidities (i.e., cross-lagged effects) were tested. The cross-lagged coefficients for the two morbidities from 14 years old (Y2: second grade of middle school) to 15 years old (Y3: third grade of middle school) were not significant for either sex. However, the CL coefficients for the two morbidities from 15 years old (Y3: third grade of middle school) to 16 years old (Y4: first grade of high school) demonstrated different patterns by sex. In males, the CL coefficients indicating that delinquency at 15 years old (Y3: third grade of middle school) accounts for depression at 16 years old (Y4: first grade of high school) were statistically significant in upward trends (*β* = 0.096, *SE* = 0.029, *p* < 0.01). Contrarily, the corresponding coefficients indicating that depression at 15 years old (Y3: third grade of middle school) accounts for delinquency at 16 years old (Y4: first grade of high school) were not significant (*β* = 0.023, *SE* = 0.030, *p* = 0.430). In females, the findings were the opposite. The CL coefficients indicating that depression at 15 years old (Y3: third grade of middle school) accounted for delinquency at 16 years old (Y4: first grade of high school) were statistically significant (*β* = 0.102, *SE* = 0.030, *p* < 0.01) with upward trends, whereas the CL coefficients indicating that delinquency at 15 years old (Y3: third grade of middle school) accounted for depression at 16 years old (Y4: first grade of high school) were not significant (*β* = −0.015, *SE* = 0.028, *p* = 0.603). In sum, males experienced delinquency before depression from when they were 15 years old (Y3: third grade of middle school) to 16 years old (Y4: first grade of high school) transition, whereas females experienced depression first and then delinquency in the same transitional period. These findings suggest that sex plays a role in the relationship between depression and delinquency.

**Table 3:** Structural model parameter estimates for the final ACLM for depression and delinquency.

			Boys (*n* = 1,062)			Girls (*n* = 1,013)		
			*B*	*SE*	*β*	*B*	*SE*	*β*
ACLM	Depression Y2 (Age 14, MS 2nd)	Depression Y3 (Age 15, MS 3rd)	0.477***	0.031	0.471***	0.619***	0.030	0.597***
	Depression Y3 (Age 15, MS 3rd)	Depression Y4 (Age 16, HS 1st)	0.349***	0.030	0.402***	0.366***	0.035	0.409***
	Delinquency Y2 (Age 14, MS 2nd)	Delinquency Y3 (Age 15, MS 3rd)	0.425***	0.025	0.461***	0.293***	0.019	0.430***
	Delinquency Y3 (Age 15, MS 3rd)	Delinquency Y4 (Age 16, HS 1st)	0.232**	0.030	0.244***	0.323***	0.030	0.335***
CL coefficient	Depression Y2 (Age 14, MS 2nd)	Delinquency Y3 (Age 15, MS 3rd)	0.021	0.012	0.050	0.003	0.007	0.012
	Depression Y3 (Age 15, MS 3rd)	Delinquency Y4 (Age 16, HS 1st)	0.009	0.012	0.023	0.024**	0.007	0.102**
	Delinquency Y2 (Age 14, MS 2nd)	Depression Y3 (Age 15, MS 3rd)	0.001	0.063	0.000	0.101	0.078	0.036
	Delinquency Y3 (Age 15, MS 3rd)	Depression Y4 (Age 16, HS 1st)	0.195**	0.058	0.096**	−0.055	0.105	−0.015

*p < 0.05, **p < 0.01, ***p < 0.001.

### 3.3. Discussion

Depression and delinquency in adolescence continuously affected the two morbidities in later years, regardless of sex. This finding supports previous studies’ findings that depression and delinquency stabilize and persist over time (46, 79). Depression is particularly more stable in adolescence than early childhood, and delinquency is more stable in adolescence than adulthood (80). Adolescence shows stronger stability for depression and delinquency than other life stages. Moreover, adolescent depression and delinquency are symptoms that manifest continuously and are associated with more serious problems later in adulthood (81–83). Therefore, early detection and intervention are important.

Depression and delinquency at 14 years old (Y2: second grade of middle school) were not causative of their manifestations at 15 years old (Y3: third grade of middle school). However, a causal relationship was established between the ages of 15 (Y3: third grade of middle school) and 16 (Y4: first grade of high school). These results may be attributed to the difference between middle school and high school years in South Korea. Especially in South Korea, where education is given a great deal of importance, high school academic performance is paramount because it is considered for university admission. Therefore, shifting from middle school to high school means being exposed to more academic-related stress than ever before, which in turn causes various mental health problems (84). Therefore, it can be assumed that problems at 16 years old (Y4: first grade of high school) become more serious because adolescents who experienced depression or delinquency at 15 (Y3: third grade of middle school) are more vulnerable to such stress (85).

Sex differences had a causal relationship with the development of depression and delinquency. In males, delinquency at 15 years old (Y3: third grade of middle school) influenced depression at 16 years old (Y4: first grade of high school). Contrarily, depression among 15-year-old females (Y3: third grade of middle school) affected delinquency at 16 years old (Y4: first grade of high school). For males, the findings are consistent with previous studies that adolescent males reflect the FM (31, 34–36, 43).

In males, delinquent behaviors resulted in failures such as rejection by significant role models and maladjustments to schooling. When repeated, such failures ultimately lead to depression. The FM appears more plausible considering previous findings that males are more negatively influenced by “branding” or labeling by others (86). Moreover, the 15-year-olds (Y3: third grade of middle school) were closely associated with the task of getting into high school; consequently, students can experience low self-confidence if their delinquent behaviors result in unsatisfactory academic outcomes. Even if they make it into high school or fail to successfully adjust to environmental changes such as higher academic pressure, the resulting lower self-confidence can lead to depression. Notably, this result was not found in the other years studied (Y2–Y3: 14–15 years), possibly because at 14 years old (Y2: second grade of middle school), delinquent behaviors and the resulting failure experiences were not serious enough to cause depression. However, more extensive studies of different age groups are needed for a definite conclusion.

Unlike males, females reflected the AOM, where internalized depressive symptoms manifest in the form of delinquency when acted out. In other words, if 15-year-old girls (Y3: third grade of middle school) experience depression, their depressive symptoms are likely to lead to delinquent behaviors at 16 years old (Y4: first grade of high school). These results supported Kofler et al.’s (87) findings that for girls (ages 12 to 17), previous depression leads to later delinquency. It is noteworthy that results of previous studies that included girls often supported the RRCP position rather than the AOM (37, 43, 45). Having said this, extensive future studies need to further investigate whether the AOM or the RRCP applies to other periods as well, with unique patterns observed for males and females. In contrast to many previous studies conducted in the West, the current study found that girls were supported for AOM rather than RRCP. The results indicate that depression might cause delinquency, but delinquency might not influence depression cyclically. For example, Korean girls often experience depression due to conflicts with their parents and tend to fall into delinquency in the process of making friends to escape from depression (86). More specifically, they might begin delinquent behavior with their friends. A peer relationship is working as a social support system to prevent depression (88). Therefore, in the case of 16-year-old girls in South Korea, the path through which delinquency causes depression becomes meaningless as the support of friends has a more important effect than the failure caused by delinquency.

Since both depression and delinquency are affected by sex, it makes sense that the relationship between them is also affected by it. Studies in various countries commonly report that women’s depression is 1.5–2 times higher than men’s (89, 90), and that the sex difference in depression symptoms has emerged since early adolescence, indicating that the gap gradually increases (91, 92). In the case of delinquency, there is no difference between men and women, but the frequency is 2–3 times higher in male adolescents than in female adolescents (93). Some studies about the relationship between delinquency and other variables have also shown sex differences. Adverse childhood experiences (ACEs) during early childhood may be implicated in boys’ delinquency, while ACEs are not significantly associated with girls’ delinquency (94). Furthermore, delinquency in males is more strongly affected by peer factors than in females (95).

Our results revealed that depression and delinquency at 15 years old (Y3: third grade of middle school) lead to depression and delinquency at 16 years old (Y4: first grade of high school). The depression and delinquency experienced by a 15-year-old (Y3: third grade of middle school) student ahead of the entrance examination can last until high school, so it can be considered a very serious and important problem. In other words, because of the nature of Korean culture, high school entrance exams are considered very important, so the stress and tension among 15-year-olds (Y3: third grade of middle school) is very high. Therefore, in the case of adolescents who experience depression or delinquency when they are 15 years old (Y3: third grade of middle school), it is necessary to intervene more urgently and sensitively in consideration of Korea’s entrance examination culture. Similar to South Korea, American teenagers also commonly identify academic stress as a significant source of stress (96). However, when examining university enrollment rates across OECD countries, the average rate is 47.08%, with the United States slightly higher at 51.17%, while South Korea boasts the highest rate at 69.3% (97). Considering these figures, it can be inferred that South Korean teenagers experience much higher levels of academic stress (98). In other words, depression and delinquency are common symptoms among American and Korean adolescents, but there may be differences in the processes leading to them. Therefore, it is important to continue further research to better understand the variations in the relationship trajectory between depression and delinquency across different age groups.

Another finding is that developmental sequences of adolescent depression and delinquency differed in males and females. Considering that few sex difference-based studies have been conducted in East Asia, the study validated a sex-based difference in the relationship between depression and delinquency in Korean adolescents. Like the major studies on boys in the West, our study on Korean boys was found to support FM. In the case of females, Western studies have mainly supported RRCP, while Korean girls supported AOM in our study. However, in past studies, there have been cases in which Korean girls supported RRCP (43), so follow-up studies are needed to understand the characteristics of Korean girls. The sex-based differences have particularly important implications for clinical interventions for the two adolescent morbidities. For example, boys experience depression resulting from various negative consequences of delinquency. Thus, immediate interventions when delinquent behavior manifests are urgently needed to prevent negative experiences from accumulating. If boys are stressed because of their failures, specific issues need to be identified to implement effective interventions. These should preferably involve positive support and assistance to ensure that boys experience increased success. For males who are already depressed, extra caution is needed because their delinquent behaviors might have fewer outward manifestations, leading to the false notion that their problems have improved. In depressed girls, it would be equally important to intervene before their morbidity develops into delinquency. However, depression is an internalized symptom that rarely manifests outwardly, making early detection harder. Moreover, girls are more prone to depression than boys (99, 100). Thus, preventive education or guidance for all girls may be beneficial to help them effectively cope with depression. Given the difficulty of early detection, active detection and support for depressed students through screening programs, parent and teacher observations, and counseling interviews are necessary. Recently, in accordance with the findings from the study, cognitive-behavioral group counseling programs based on mindfulness, such as mindfulness-based stress reduction (MBSR) and mindfulness-based cognitive therapy (MBCT) have been widely used to effectively reduce depression (101). As treatments for depressed girls continue to be discovered (101–103), it would be helpful to design treatments for the school context.

This study has limited applicability to varied age groups. Future studies should address adolescents with a wider range of demographic characteristics. Since the participants were school-educated adolescents, sampling and measurement may have excluded those with serious or major problems (such as out-of-school adolescents). Therefore, future studies should be expanded to include more vulnerable, high-risk adolescents. The adolescents’ panel data are self-reports, possibly resulting in response bias. Further, different levels of delinquency are influenced by different factors (104, 105). Apart from the advantage of conducting research using open data provided by public institutions in the information society, there is a limit to the fact that the scale of variables cannot be planned and designed directly. Depending on the delinquency level, the relationship between delinquency and depression varies. Moreover, future studies should differentiate delinquency levels in their design.

## Data availability statement

Publicly available datasets were analyzed in this study. This data can be found at: https://www.nypi.re.kr/archive/mps.

## Ethics statement

The studies involving human participants were reviewed and approved by the Institutional Review Board (IRB) for human subjects of Dongguk University (DUIRB-202208-08). Written informed consent from the participants’ legal guardian/next of kin was not required to participate in this study in accordance with the national legislation and the institutional requirements.

## Author contributions

NK, YJ, and YIC: study concept and design and statistical analysis and interpretation of data. NK, YJ, YIC, YYC, and SP: writing–original draft and access to data. YIC, YYC, and SP: study supervision. All authors contributed to the article and approved the submitted version.

## Funding

This work was supported by the National Research Foundation of Korea (NRF) grant funded by the Korean government (MIST) (No. 2018R1A5A7023490).

## Conflict of interest

The authors declare that the research was conducted in the absence of any commercial or financial relationships that could be construed as a potential conflict of interest.

## Publisher’s note

All claims expressed in this article are solely those of the authors and do not necessarily represent those of their affiliated organizations, or those of the publisher, the editors and the reviewers. Any product that may be evaluated in this article, or claim that may be made by its manufacturer, is not guaranteed or endorsed by the publisher.
